# Supplementary material for: Synthesis and characterization of selenium nanoparticles stabilized with oxyethylated alkylphenol (neonol) for potential modification of fabric materials
Source: PLoS One. 2024 Nov 26;19(11):e0314208. doi: 10.1371/journal.pone.0314208 (PMC11593756; doi:10.1371/journal.pone.0314208)
Supplement: Attachment — Submitted filename: Response to Reviewer.docx [file pone.0314208.s002.docx]

Reviewer #1: My second comment was not addressed, so I repeat it.

2. How the authors would explain the antimicrobial activity of SeNP-modified fabric

materials if nanoparticles are strongly immobilized on their surfaces, thus hindering the

interactions with bacterial cells? Some positive and negative controls should be

performed with SeNPs and untreated fabric materials.

Response: Thank you for the comment! The article describes the microbiological study and the results obtained during the experiment. Even though selenium nanoparticles are included on the surface of the suture material, selenium still exhibits antimicrobial activity. We have added information on the probable mechanism of action of selenium nanoparticles. Detailed analysis of the mechanism of action of selenium nanoparticles on microorganisms is planned for future works.

New changes are marked by red.

Reviewer #2: (No Response)

Reviewer #3: The authors have adequately addressed reviewer's comments and therefore it is suitable for publication.
